# Supplementary material for: H3 ubiquitination by NEDD4 regulates H3 acetylation and tumorigenesis
Source: Nat Commun. 2017 Mar 16;8:14799. doi: 10.1038/ncomms14799 (PMC5357315; doi:10.1038/ncomms14799)
Supplement: Supplementary Information — Supplementary Figures [file ncomms14799-s1.pdf]

a

Protein name	Ubiquitination site	Pubmed ID
Histone H3.3	K14, K18, K23, K27, K36, K56, K79, K122	21890473, 21906983
histone H3.3C	K14, K18, K23, K56, K122	21890473, 21906983, 20972266
Histone H3	K18, K23, K56, K79	21890473

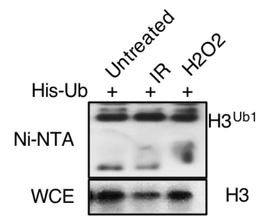

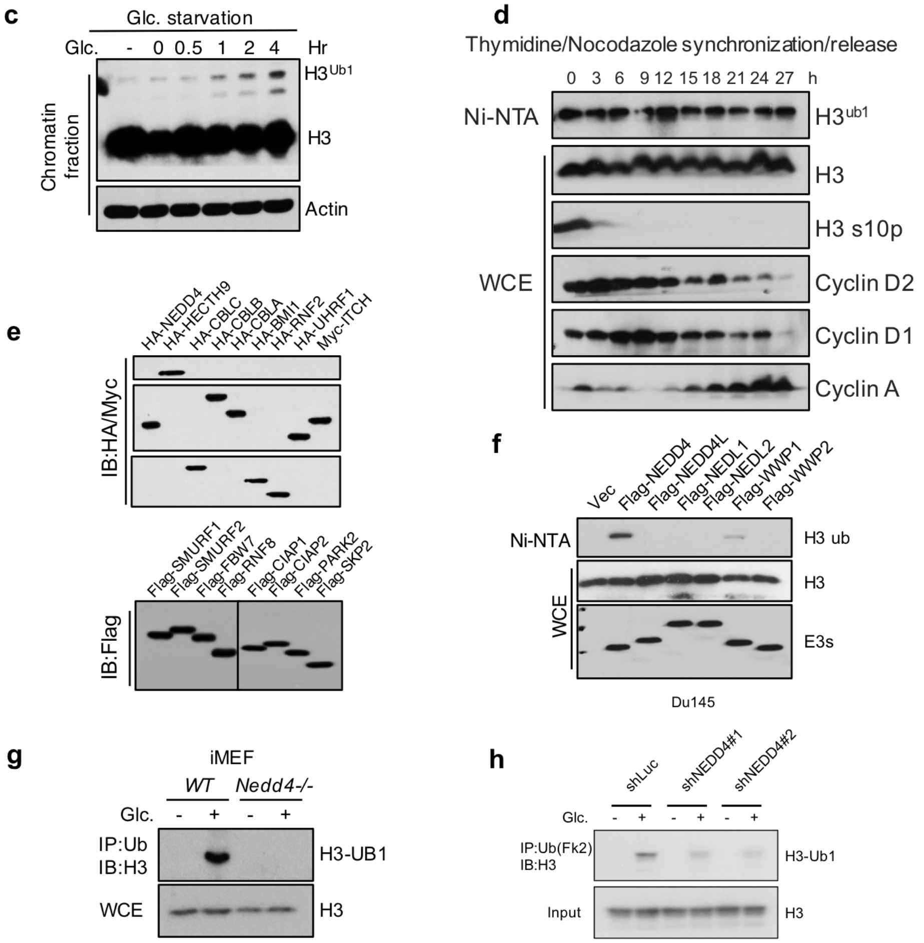

Supplementary Fig. 1 a. Shown is a table view of H3 ubiquitination sites identified in large-scale quantitative proteomics studies. b. 293T cells were transfected with his-ubiquitin plasmid (His-Ub) for 36 hours and treated with various stresses for 4 hours before *in vivo* ubiquitination assay. c. Glucose induces H3 ubiquitination in a time dependent manner. Hep3B cells were glucose starved for 4 hours and added-back glucose for indicated time before chromatin fractionation assay. d. 293T cells transfected with his-Ub plasmid was synchronized by sequential treatment of Thymidine (2mM, 24h) and Nocodazole (100ng/ml, 12h). In vivo ubiquitination assay was then performed to assay the impact of cell cycle on H3 ubiquitination. e and f. E3 transfection level control for Fig. 1D. Whole cell lysate was analyzed by Western blot. g and h. WT/NEDD4-/- immortalized MEFs (iMEFs) or Control/NEDD4 knockdown Du145 cells were treated with glucose and harvested for immunoprecipitation assay.

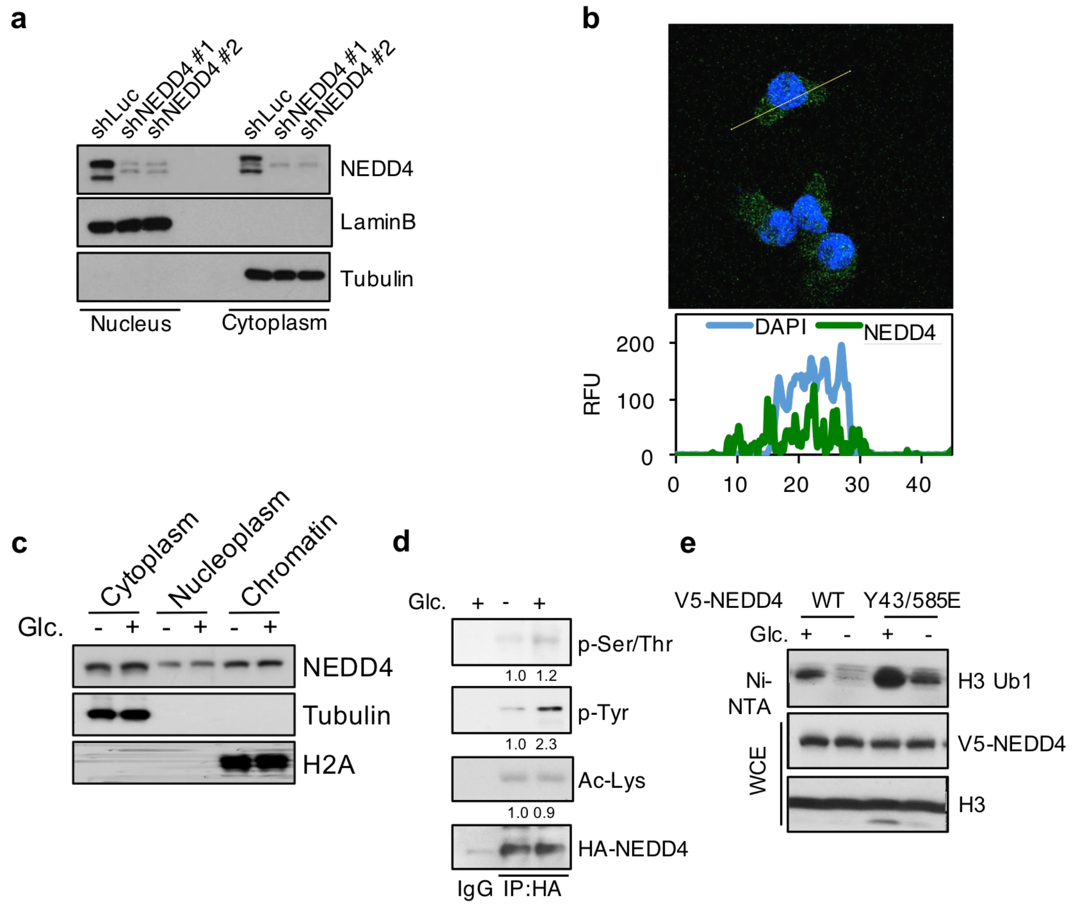

Supplementary Fig. 2 a. Cellular fractionation was performed for control and NEDD4 knockdown Hep3B cells. b. Immunofluorescence assay was performed for Hep3B cells. The intensity of NEDD4 staining (Green) and DAPI staining (Blue) along the yellow line in the image was shown in the histogram. c. Chromatin fractionation was performed for the Hep3B cells treated with or without glucose. d. 293T cells transfected with HA-NEDD4 was treated with glucose and harvested for immunoprecipitation assay. e. 293T cells were transfected with His-Ub and indicated plasmids and treated with glucose before *in vivo* ubiquitination assay.

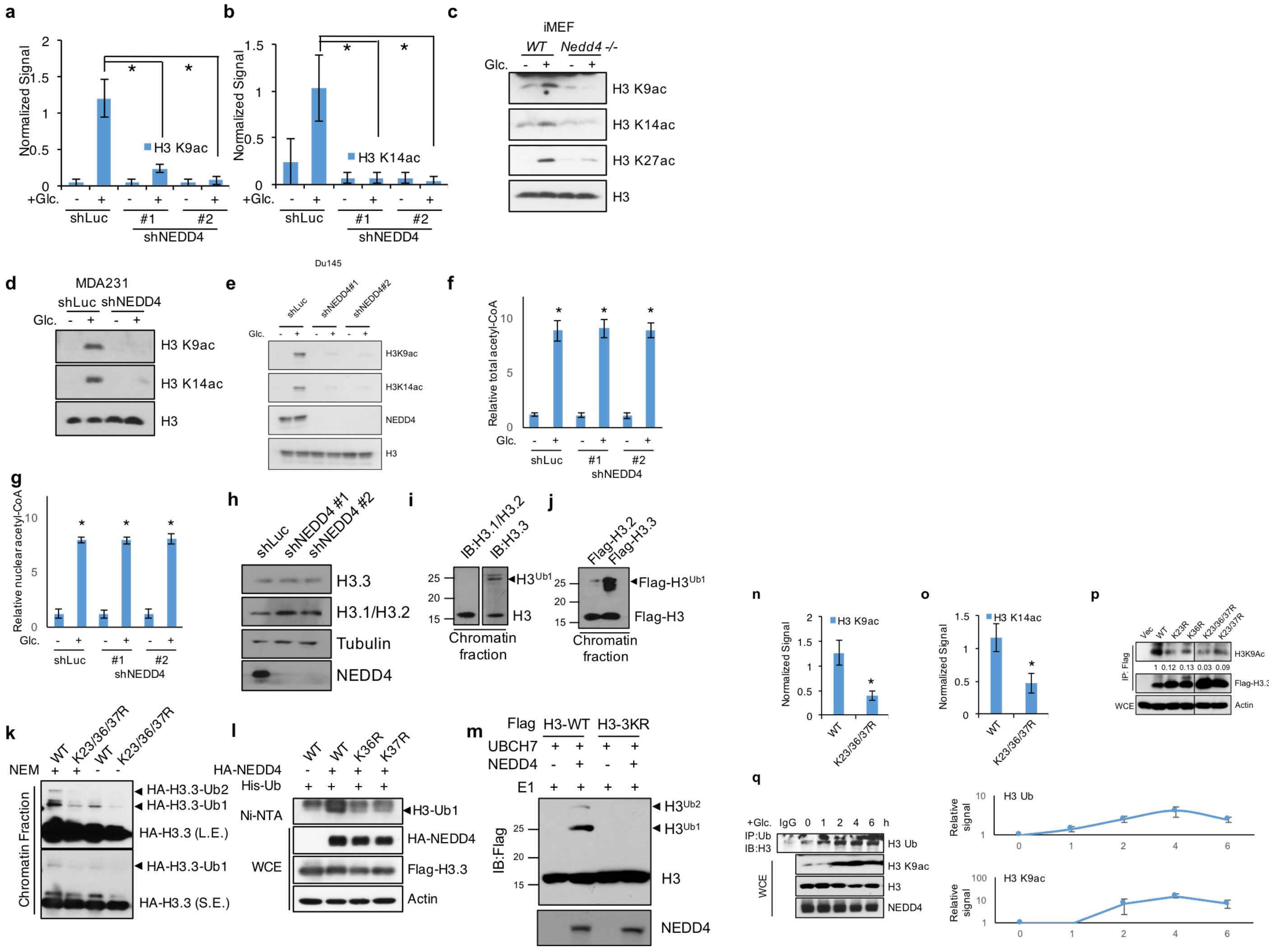

Supplementary Fig. 3 a and b. Quantification of Western blot data (n=3, mean ± s.d.). c, d and e. NEDD4 deficient iMEF cells, MDA231 or Du 145 cells were treated with glucose and harvested for Western blot analysis. f and g. NEDD4 does not affect glucose feeding induced elevation of acetyl-CoA level. Control and NEDD4 knockdown Hep3B cells were glucose starved and fed with glucose before measuring acetyl-CoA level by PicoProbe assay kit (Biovision). h. H3.3 and H3.2/H3.1 antibody was assayed for control and NEDD4 knockdown Hep3B cells. i. Chromatin fractionation from Hep3B cells was blotted for H3.1/3.2 and H3.3. j. Chromatin fractionation assay was performed by 293T cells expressing Flag-H3.2 and Flag-H3.3. k. Chromatin fractionation was performed for Hep3B cells stably expressing Flag-H3.3 WT or K23/36/37R. NEM was added to preserve ubiquitination. l. *In vivo* ubiquitination assay was performed for 293T cells, which are transfected with indicated plasmids. m. In vitro ubiquitination assay was performed for Flag-H3-WT and Flag-H3-K23/36/37R containing nucleosomes (See methods for details). n and o. Quantification of Western blot data (n=3, mean ± s.d.). p. Various mutants of Flag-H3.3 were restored in shH3.3 Hep3B cells. Immunoprecipitation assay was used to access the H3 K9ac. q. Hep3B cells treated with glucose for various times (hours) were harvested for immunoprecipitation assay. Kinetics of H3 ubiquitination and K9ac were shown in log scale and plotted as the average of three independent biological repeats.

All asterisks (*) represent $p<0.05$, using Student's T test.

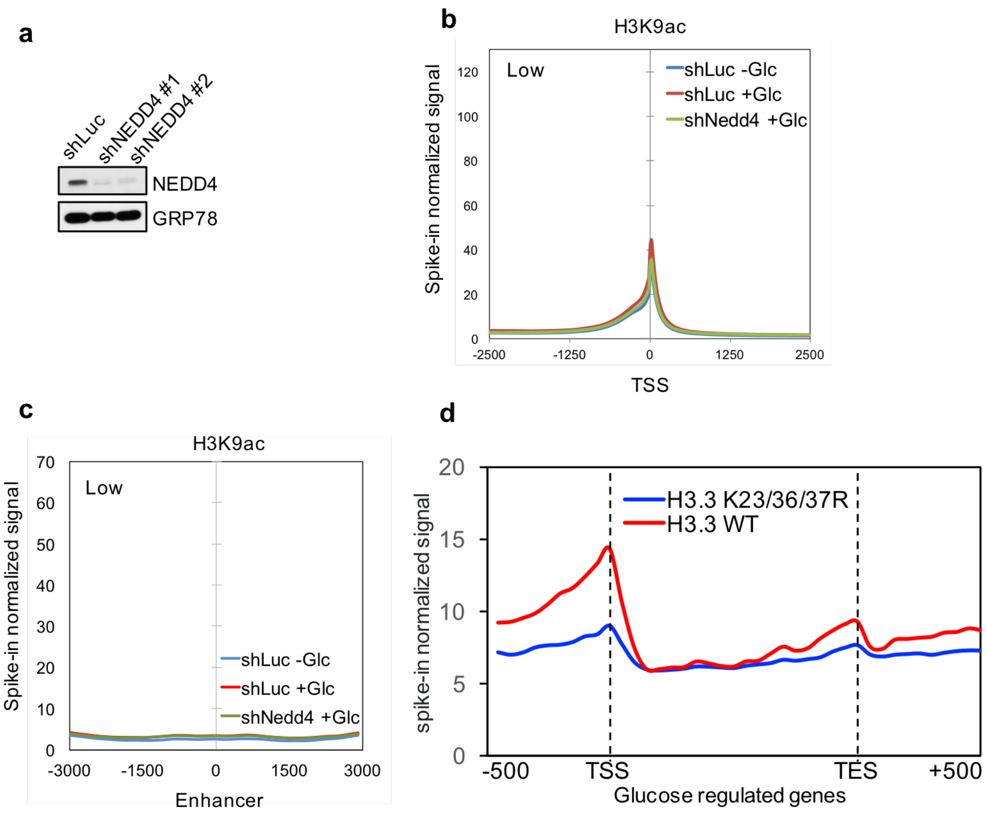

Supplementary Fig. 4. a-c. Shown is NEDD4 knockdown efficiency in Hep3B cells (a), global profile of H3 K9ac at TSS for genes with low H3 K9ac (b), and global profile of H3 K9ac at enhancers, which have low H3 K9ac (c). d. ChIP-seq assay using anti-conjugated ubiquitin antibody (FK2) was performed for Hep3B cells restored with H3.3 WT and H3.3 K23/36/37R. Shown is the profile of the occupancy of ubiquitinated proteins for glucose upregulated genes (genes with glucose inducible H3K9ac derived from fig. 3b) from -500bps of TSS to +500bps of TES.

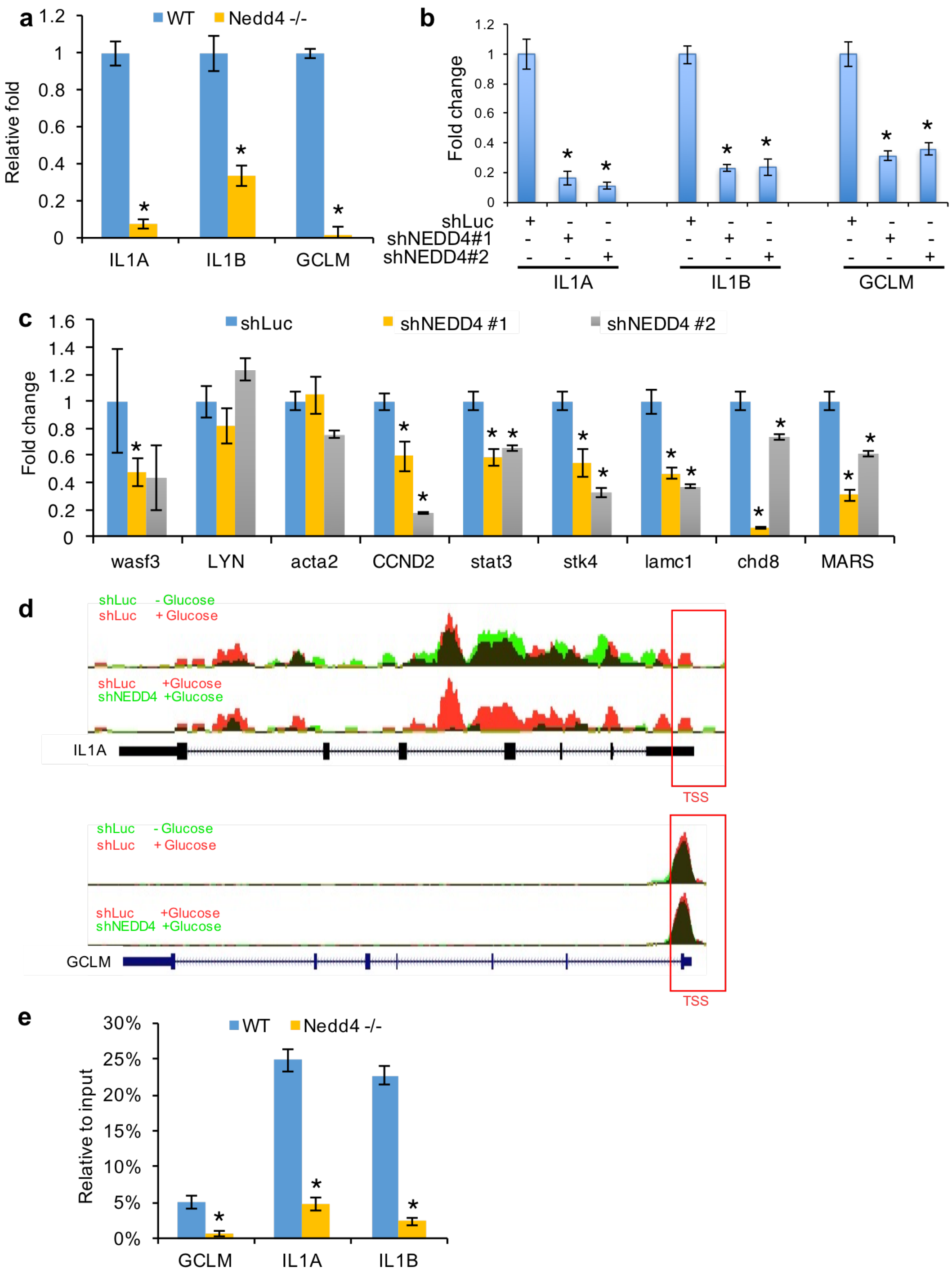

Supplementary Fig. 5 a, b and c. qPCR was performed to analyze the mRNA level in NEDD4 deficient iMEFs (a), Du 145(b) or Hep3B(c) cells (n=3, mean ± s.e.m.). d. UCSC genome browser view of ChIP-seq H3 K9ac signals along IL1A and GCLM genes. e. ChIP-qPCR was performed for WT and Nedd4-/- iMEFs.

All asterisks (*) represent $p<0.05$, using Student's T test.

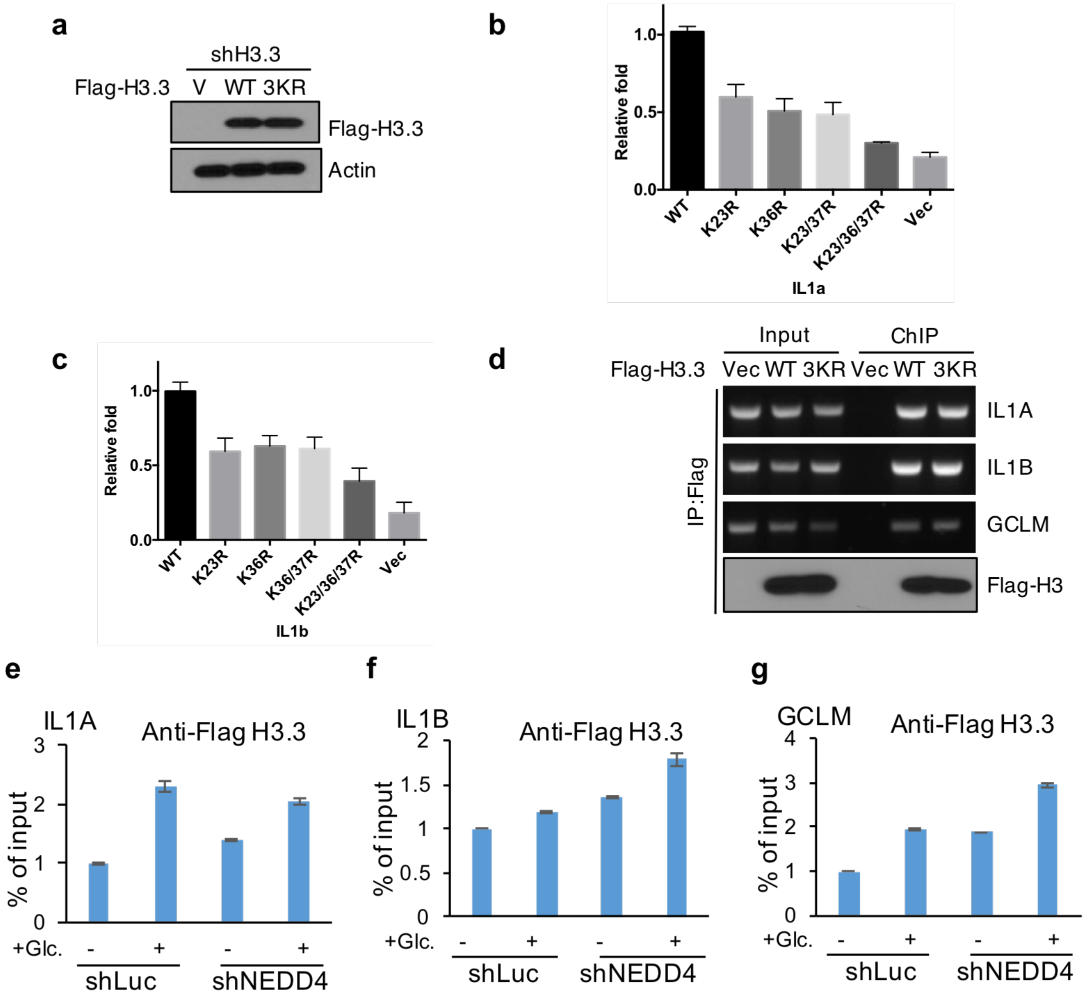

Supplementary Fig. 6 a. Shown are Flag-H3.3 WT and K23/36/37R restoration efficiency in H3.3 knockdown Hep3B cells. b and c. qPCR was used to analyze IL1a and IL1b mRNA level in Hep3B cells restored with various H3.3 constructs. d. ChIP-qPCR assay was performed for Flag-H3.3 WT and 3KR mutant. e-g. ChIP-qPCR was performed for control and NEDD4 knockdown Hep3B cells.

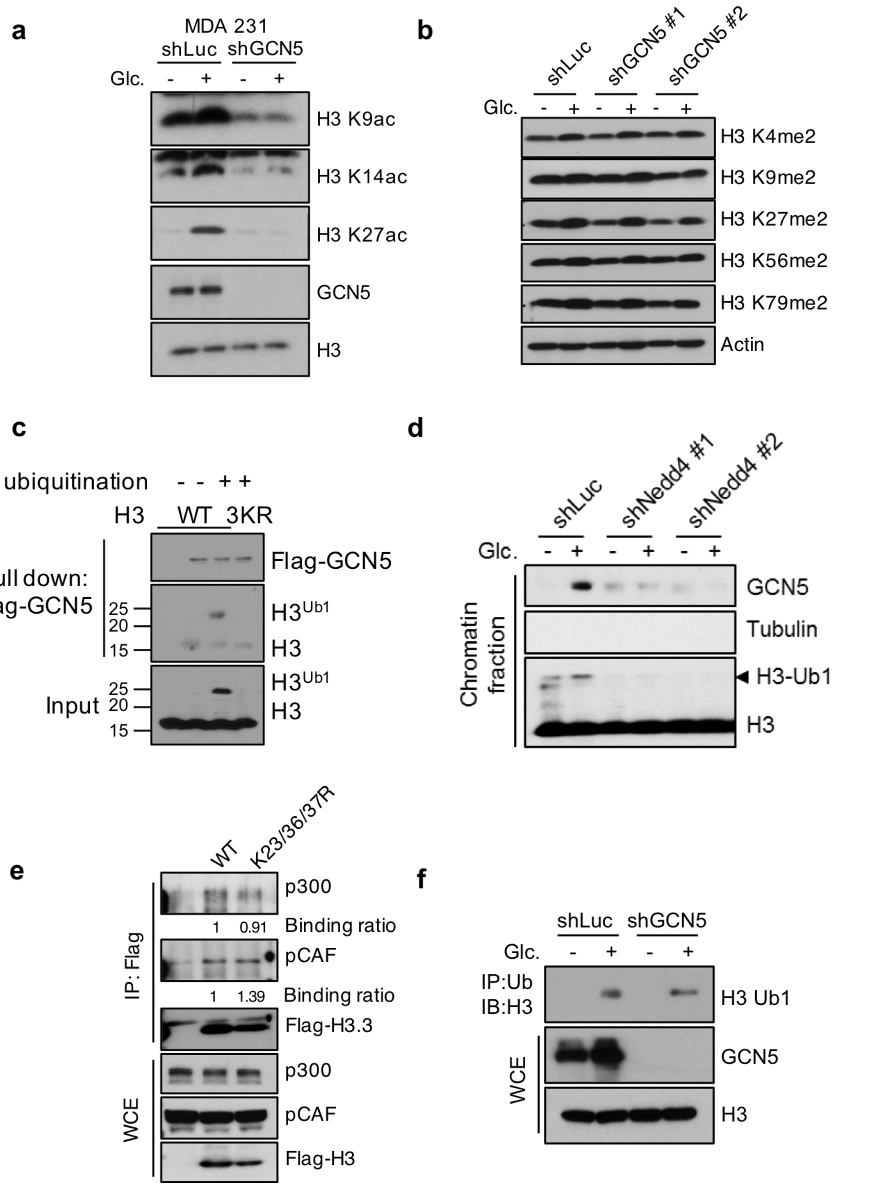

Supplementary Fig. 7 a. Control and GCN5 knockdown MDA 231 cells were treated with glucose and harvested for Western blot analysis. b. GCN5 is not required for H3 di-methylation. Control and GCN5 knockdown Hep3B cells were glucose-starved for 4 hours and added-back glucose for 2 hours before whole cell extraction for Western blot analysis. c. In vitro binding assay was performed for purified Flag-GCN5 containing complex from 293T cells and in vitro ubiquitinated WT and 3KR containing nucleosomes purified from 293T cells (See methods for details). Of note, NEM is not used in the purification of Flag-GCN5 to prevent co-purification of endogenous ubiquitinated H3. d. Chromatin fractionation was performed for control and NEDD4 knockdown cells. e. H3 ubiquitination on K23/36/37 sites is not required to recruit p300 or pCAF to histone H3.3. Stably expressed Flag-H3.3 WT or K23/36/37R was immunoprecipitated from Hep3B cells to analyze its co-immunoprecipitates by Western blot. f. GCN5 knockdown Hep3B cells were subjected to endogenous ubiquitination assay.

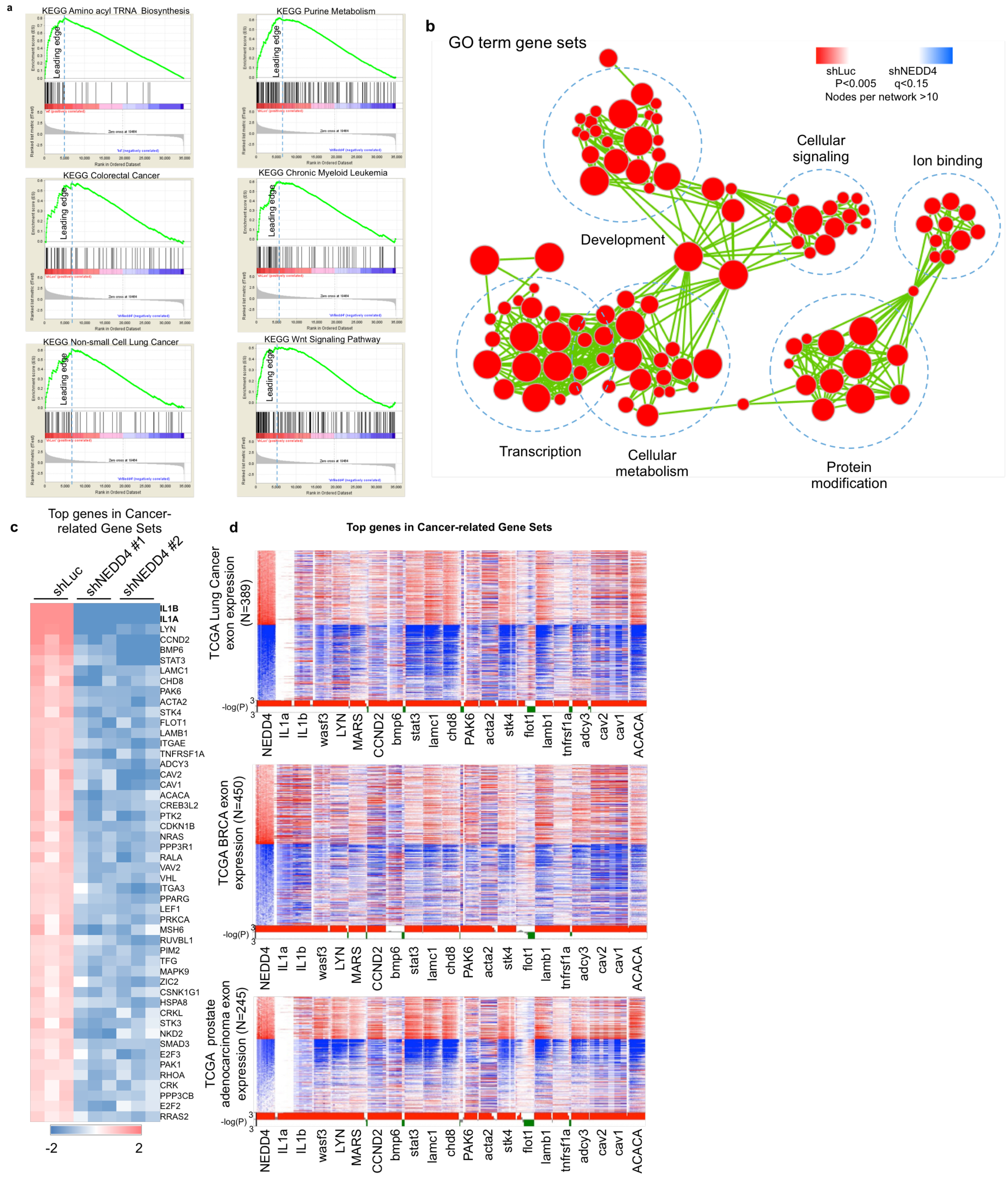

Supplementary Fig. 8 a. Multiple functional gene sets are enriched in control versus NEDD4 knockdown Hep3B cells. b. Enrichment map view of gene set enrichment analysis results. See experimental procedures for detail. c. Heat map view of top gene list of cancer-related gene sets in Fig. 6a. d. Heat map view of the TCGA exon expression profile for the NEDD4 and NEDD4 target genes from cancer-related gene sets. Patient samples ranked tops or bottoms 30% for NEDD4 expression were included and rank listed. Wilcoxon test was performed to evaluate the correlation of gene expression between NEDD4 and each gene. Significant ($p<0.01$) positive correlations were shown in red and negative correlations were show in green. See experimental procedures for details.

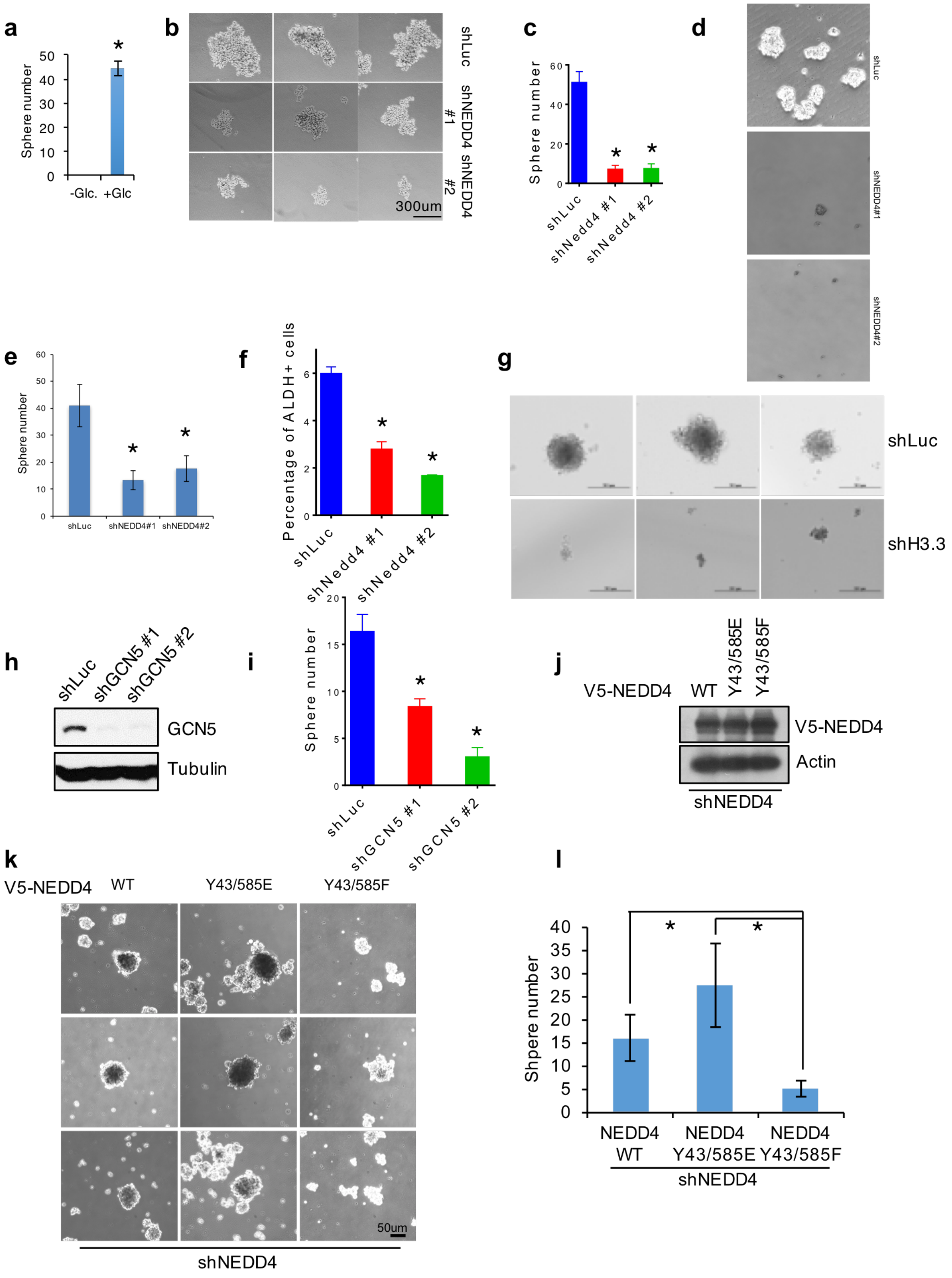

Supplementary Fig. 9 a. Glucose deprivation abolished *in vitro* tumour sphere formation for Hep3B cells. b-e. NEDD4 knockdown reduces *in vitro* tumour sphere number in MDA231(b, c) and Du 145(d, e) cells. f. NEDD4 knockdown reduces Aldh^{+} population in MDA231 cells. g. H3.3 knockdown reduces *in vitro* tumour sphere number in Hep3B cells. h. Shown was the GCN5 knockdown efficiency by Western blot. i. GCN5 knockdown reduces *in vitro* tumour sphere number in Hep3B cells. j-l. NEDD4 knockdown Hep3B cells transfected with WT, Y43/585E or Y43/585F NEDD4 plasmids were used for tumour sphere forming assay. Data were presented as the mean number of three biological replicates ± s.e.m.

All asterisks (*) represent p<0.05, using Student's T test.

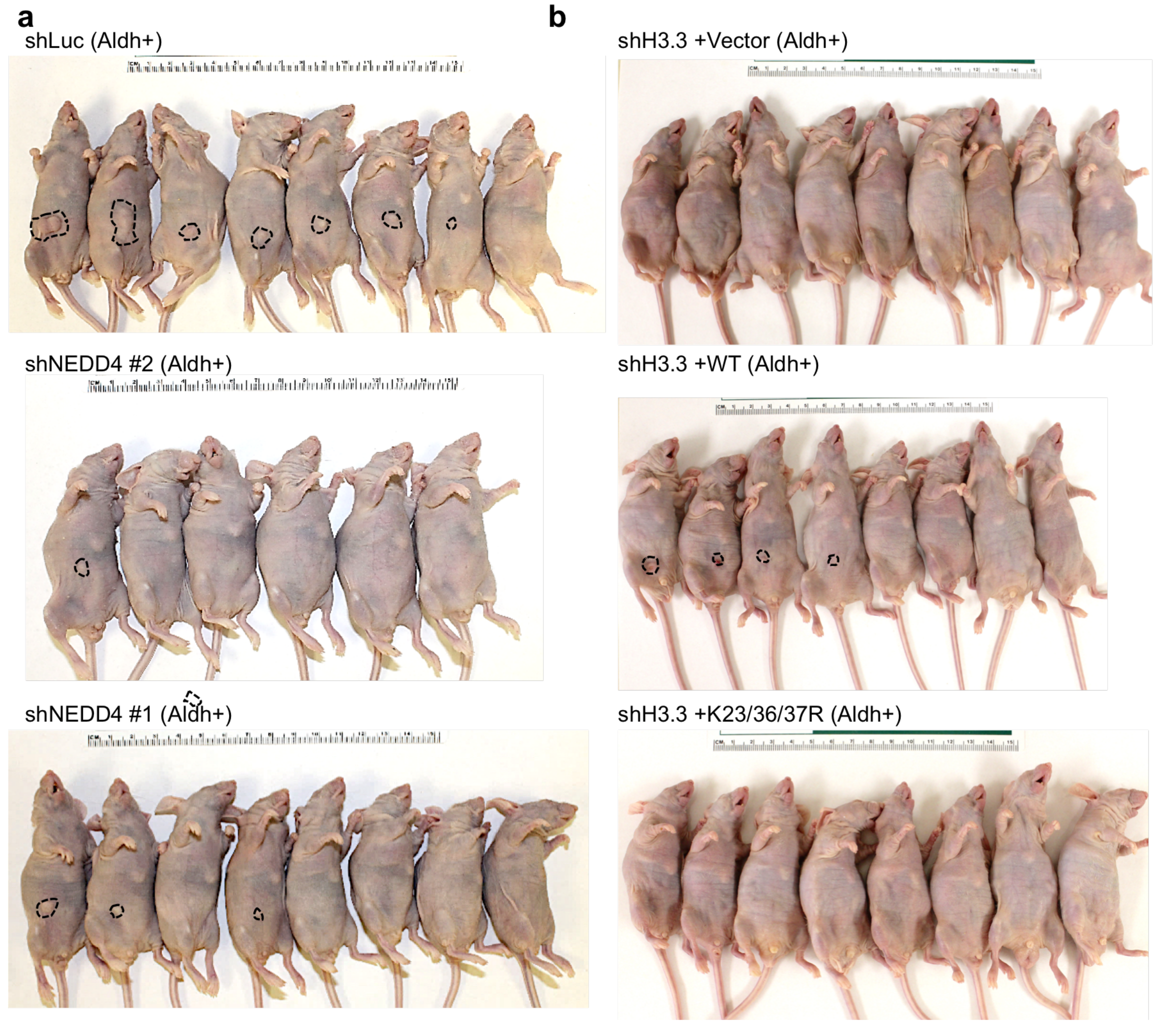

Supplementary Fig. 10 a and b. Shown are the mice image of xenograft model in fig. 6i and 6l.

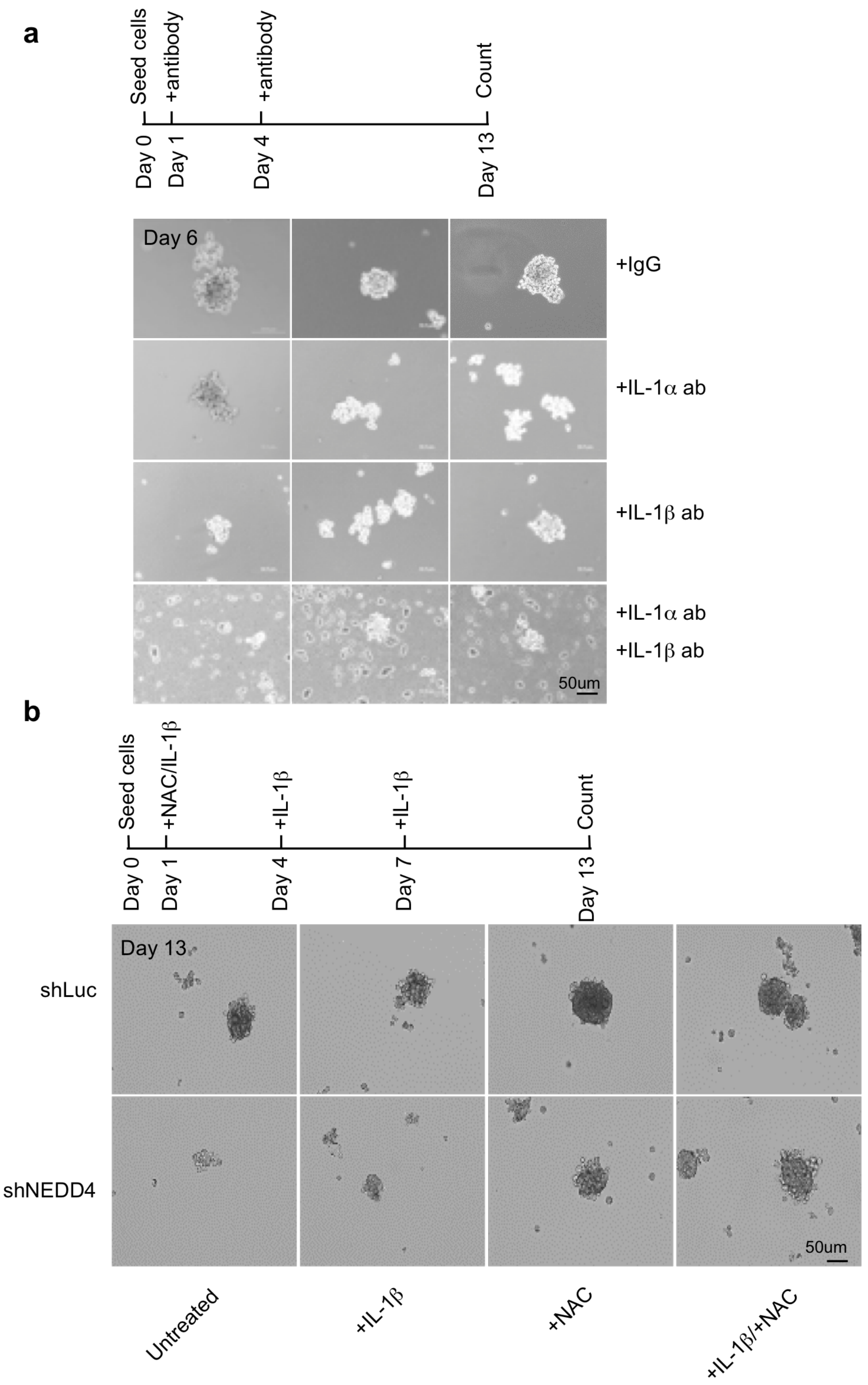

Supplementary Fig. 11 a and b. Treatment schemes and images of tumour sphere formation assay in Fig. 7a and 7b. See experimental procedures for details.

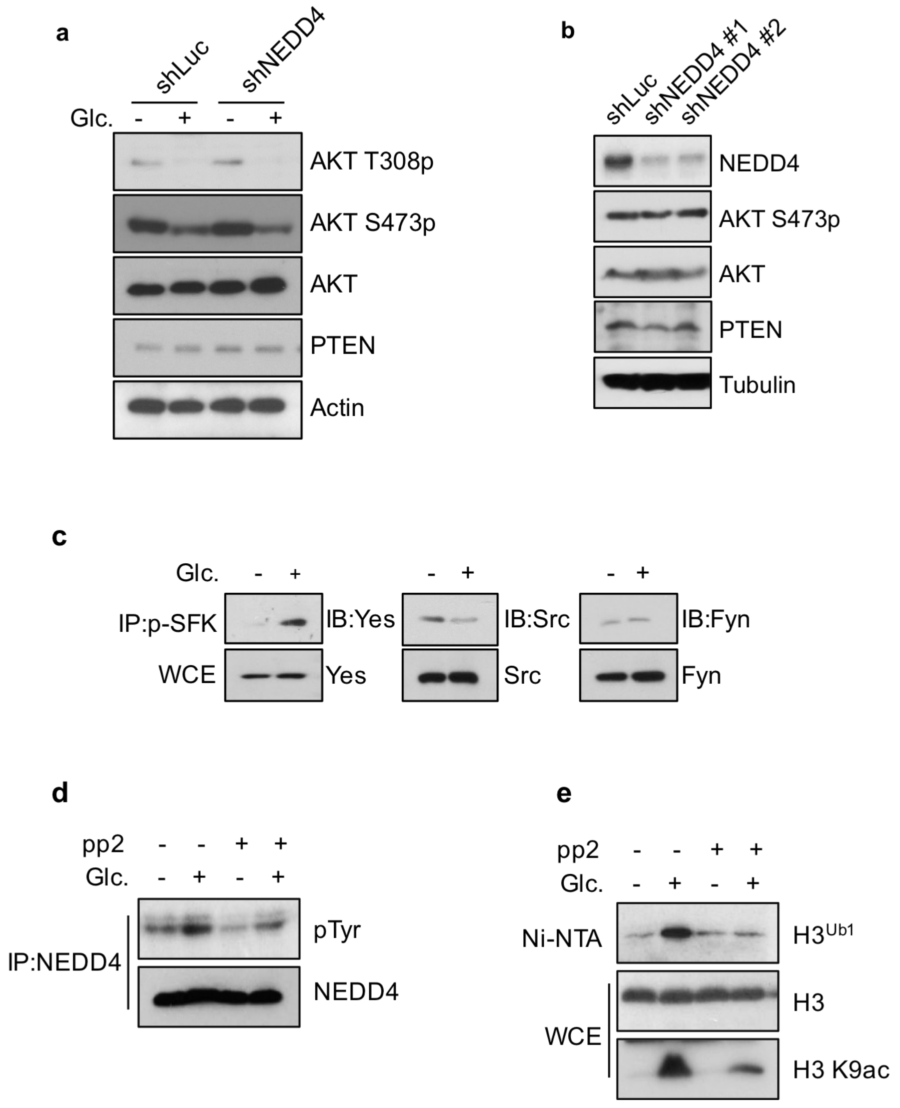

Supplementary Fig. 12 a. Hep3B cells treated with or without glucose were analyzed by Western blot. b. Cancer spheres from fig. 6c were analyzed by Western blot. c. Hep3B cells were glucose starved and treated with glucose for 1h before immunoprecipitation assay. p-SFK (Cell signaling #2101) antibody detects the consensus active phosphorylation of Src family kinases. d. Src family kinase (SFK) inhibitor PP2 was pretreated for 1h before glucose add-back and immunoprecipitation assay was performed to access the tyrosine phosphorylation of NEDD4. e. PP2 was pretreated for 2h before glucose add-back and in vivo ubiquitination assay was performed to access the H3 ubiquitination.

Fig. 1c

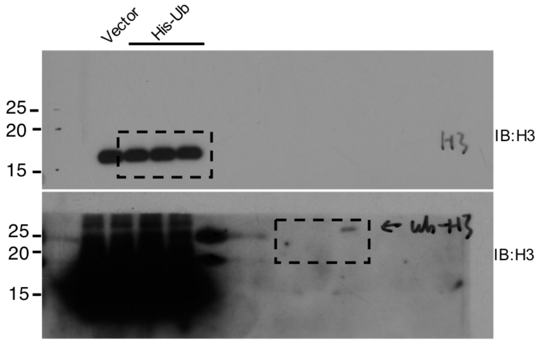

Fig. 1d

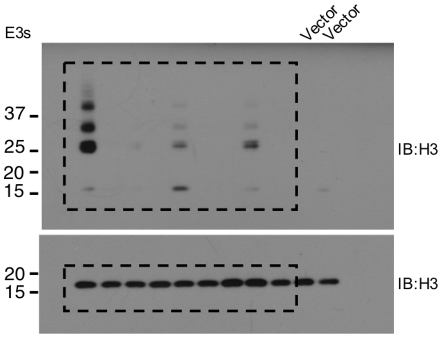

Fig. 1d

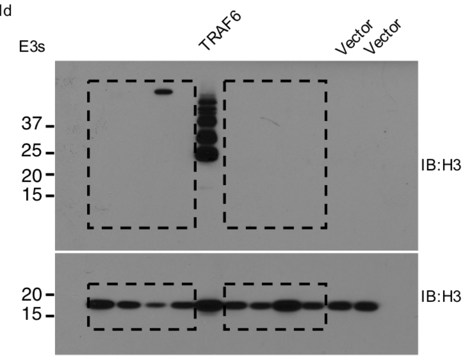

Fig. 1e

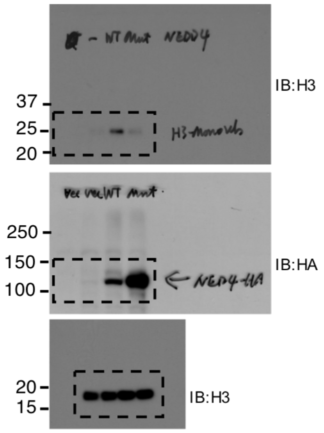

Fig. 1g

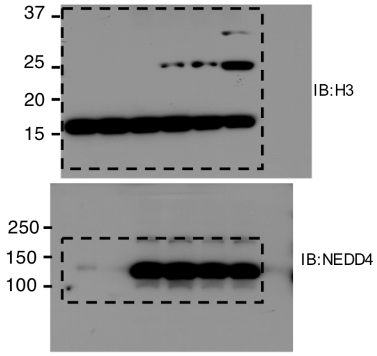

Fig. 1h

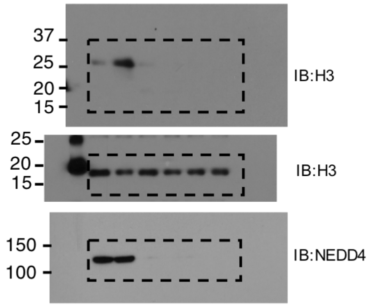

Fig. 1j

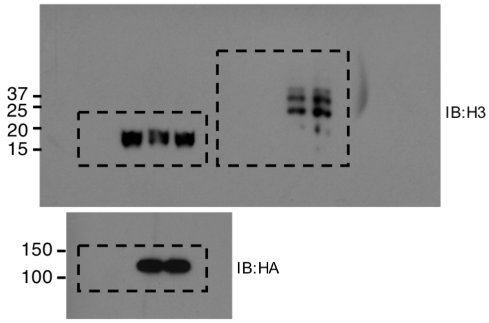

Fig. 1k

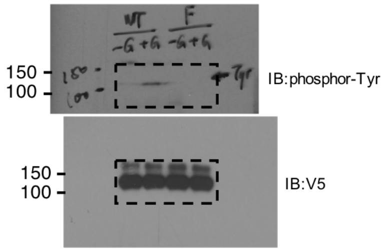

Fig. 1l

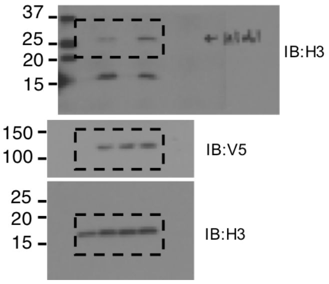

Fig. 2a

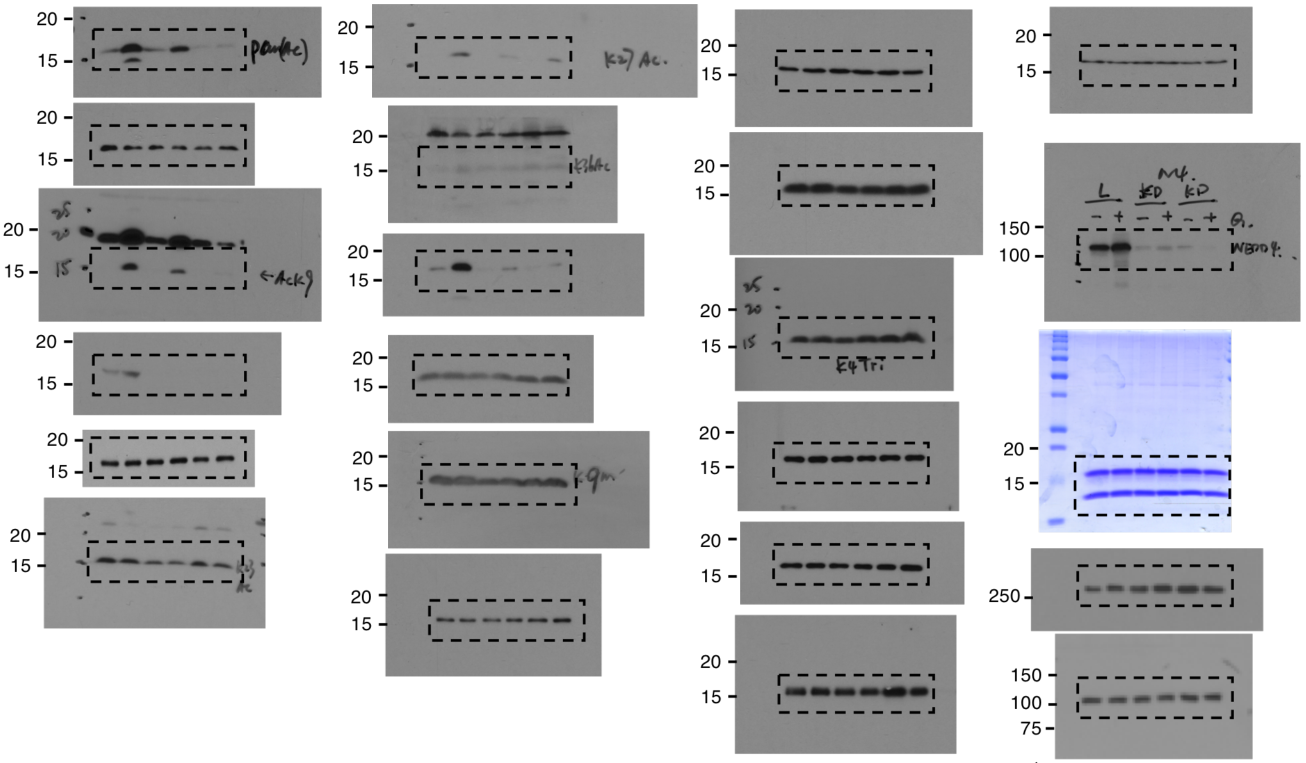

Fig. 2c

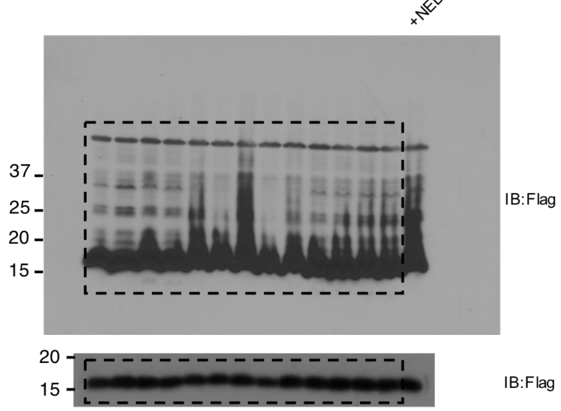

Fig. 2d

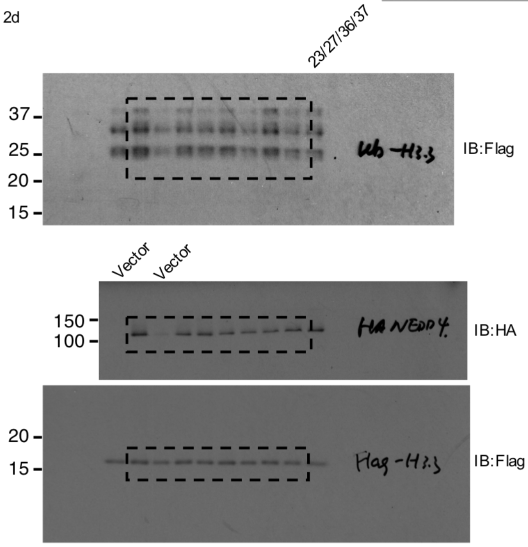

Fig. 2e

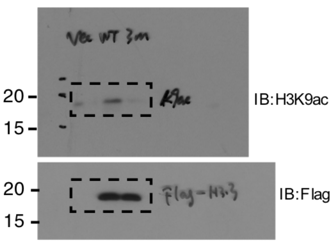

Fig. 2f

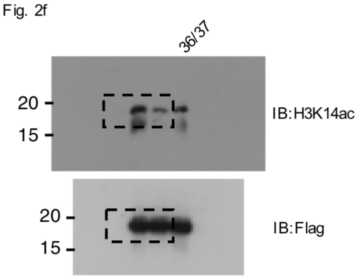

Fig. 2g

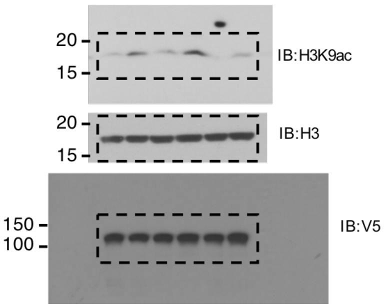

Fig. 5g

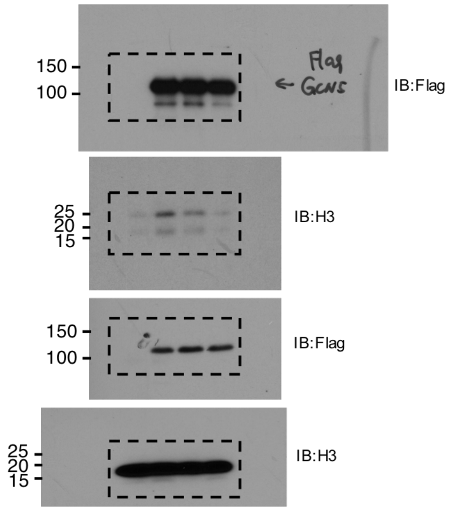

Fig. 5h

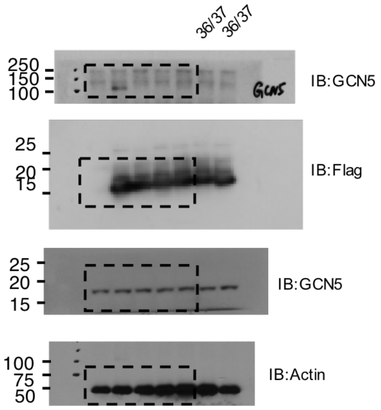

Fig. 5f

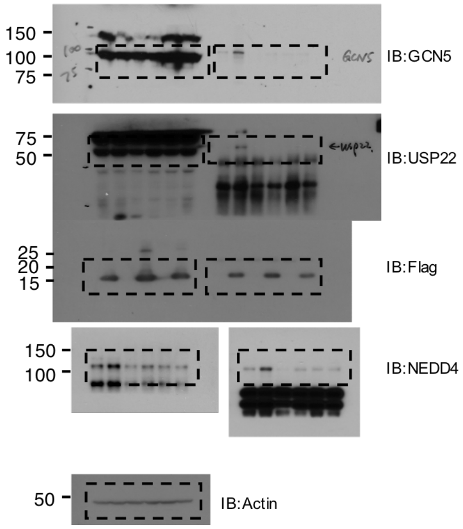

Fig. 5i

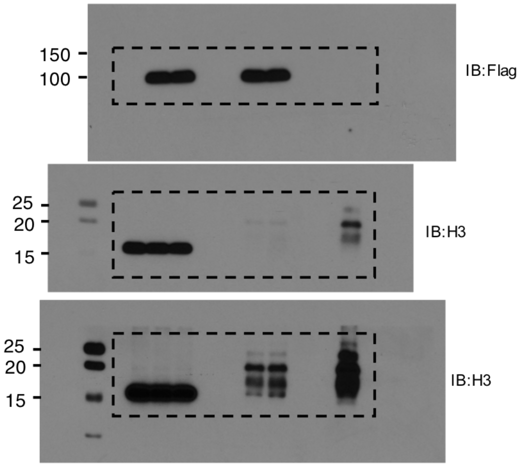

Supplementary Fig. 13 Uncropped scans for Western blot data in main figures.
